# Tokology

**Published:** 1889-12

**Authors:** 


### TOKOLOGY.

The above is the title of a most excellent work which has attracted wide-spread attention, relating as it does to the science of reproduction from a purely physical stand-point, and conveying a species of information not usually attainable in a popular treatise, in plain but acceptable terms. Its author is the head of the publication house of Alice B. Stockham & Co., 161 La Salle Street, Chicago, a physician and writer of known ability, and one of the chief contributors of *The Kindergarten*, a monthly published by this firm, which has now reached No. 8 of its second year.

Dr. Stockham is at present on a visit of business and pleasure abroad, and it is gratifying to her friends to know that she has been everywhere received with marked attention. She is the first American woman ever honored by a reception given by the Woman's Club of Helsingfors, Finland. The President of this club, the Baroness Gripenberg, is to translate Tokology into Swedish. Dr. Stockham's departure for St. Petersburg, was an ovation. The comments on her bravery in venturing alone into the country of the Czar were received by her with her wonted smiling composure.

She found her presence much needed in Moscow, where the translation of Tokology into the Russian language is being effected under the able supervision of Count Tolstoi. Within a year this grand book can be read in four languages. Thus it is truly a boon to *every* woman. It will soon, like a life-giving, health-bringing girdle, encircle the world. Every woman in every clime may in it find a blessing.

For sale by Alice B. Stockham & Co., Chicago.

---

HASTE AND HEALTH.—Nowadays, men begin to die before they learn how to live. There are no more Methuselahs. The race is being "railroaded" along at steam speed. People are too hurried to think of health—are under too much pressure to pause for physiology. They bolt their meals, race for the trains, jump for the boat. Those who live fast do not live well. The steady, moderate, methodical man does more work and better than one who tries to do in a day the work of a week. The racer gives out sooner than the plodding draught-horse. There is nothing which can be won by work in this world that can make amends for shortened and enfeebled lives. Yet there are people who take things too easy to even keep in good health. There is nothing that shortens life like laziness. A certain amount of nervous stimulous is needful for longevity. A person's life may be so peaceful, and his cares so few as to vitiate his vitality. He may decay and become prematurely old from sheer lack of employing his energies, while an active, bustling, business man may be in the full plenitude of his strength. An extreme of rust is as injurious as one of rush.

